# Tissue-Specific Ferritin- and GFP-Based Genetic Vectors Visualize Neurons by MRI in the Intact and Post-Ischemic Rat Brain

**DOI:** 10.3390/ijms21238951

**Published:** 2020-11-25

**Authors:** Marina Y. Khodanovich, Andrey E. Akulov, Tatyana V. Anan’ina, Marina S. Kudabaeva, Anna O. Pishchelko, Elena P. Krutenkova, Nikolay M. Nemirovich-Danchenko, Mikhail V. Svetlik, Yana A. Tumentceva, Chris Van den Haute, Rik Gijsbers, Veronique Daniëls, Irina Thiry, Alexandra G. Pershina, Maria M. Shadrina, Anna V. Naumova

**Affiliations:** 1 Laboratory of Neurobiology, Research Institute of Biology and Biophysics, Tomsk State University, 36, Lenina Ave., 634050 Tomsk, Russia; tany_a@list.ru (T.V.A.); kmsra08@gmail.com (M.S.K.); bio_1979@mail.ru (A.O.P.); len--k@yandex.ru (E.P.K.); nmn-d@mail.ru (N.M.N.-D.); mihasv@gmail.com (M.V.S.); mimizyana@gmail.com (Y.A.T.); m.m.shadrina@list.ru (M.M.S.); nav@uw.edu (A.V.N.); 2 Institute of Cytology and Genetics, Siberian Branch of the Russian Academy of Sciences, 10, Lavrentyeva Ave., 630090 Novosibirsk, Russia; akulov.mri@gmail.com; 3 Laboratory for Viral Vector Technology & Gene Therapy, Leuven Viral Vector Core, KU Leuven, 13, Oude Markt, 3000 Leuven, Belgium; chris.vandenhaute@kuleuven.be (C.V.d.H.); rik.gijsbers@kuleuven.be (R.G.); veronique.daniels@kuleuven.be (V.D.); irina.thiry@kuleuven.be (I.T.); 4 Central Research Laboratory, Siberian State Medical University, 2, Moskovsky st., 634050 Tomsk, Russia; allysyz@mail.ru; 5 Department of Radiology, University of Washington, 850 Republican St., Seattle, WA 98109, USA

**Keywords:** ferritin, molecular imaging, MRI, neurogenesis, adeno-associated viral vectors, gene reporters, MCAO, focal ischemia, animal models, inflammation

## Abstract

(1) Background: Neurogenesis is considered to be a potential brain repair mechanism and is enhanced in stroke. It is difficult to reconstruct the neurogenesis process only from the histological sections taken from different animals at different stages of brain damage and restoration. Study of neurogenesis would greatly benefit from development of tissue-specific visualization probes. (2) Purpose: The study aimed to explore if overexpression of ferritin, a nontoxic iron-binding protein, under a doublecortin promoter can be used for non-invasive visualization of neurogenesis using magnetic resonance imaging (MRI). (3) Methods: Ferritin heavy chain (FerrH) was expressed in the adeno-associated viral backbone (AAV) under the doublecortin promoter (pDCX), specific for young neurons, in the viral construct AAV-pDCX-FerrH. Expression of the enhanced green fluorescent protein (eGFP) was used as an expression control (AAV-pDCX-eGFP). The viral vectors or phosphate-buffered saline (PBS) were injected intracerebrally into 18 adult male Sprague–Dawley rats. Three days before injection, rats underwent transient middle-cerebral-artery occlusion or sham operation. Animals were subjected to In vivo MRI study before surgery and on days 7, 14, 21, and 28 days after injection using a Bruker BioSpec 11.7 T scanner. Brain sections obtained on day 28 after injection were immunostained for ferritin, young (DCX) and mature (NeuN) neurons, and activated microglia/macrophages (CD68). Additionally, RT-PCR was performed to confirm ferritin expression. (4) Results: T2* images in post-ischemic brains of animals injected with AAV-pDCX-FerrH showed two distinct zones of MRI signal hypointensity in the ipsilesioned hemisphere starting from 14 days after viral injection—in the ischemic lesion and near the lateral ventricle and subventricular zone (SVZ). In sham-operated animals, only one zone of hypointensity near the lateral ventricle and SVZ was revealed. Immunochemistry showed that ferritin-expressing cells in ischemic lesions were macrophages (88.1%), while ferritin-expressing cells near the lateral ventricle in animals both after ischemia and sham operation were mostly mature (55.7% and 61.8%, respectively) and young (30.6% and 7.1%, respectively) neurons. RT-PCR confirmed upregulated expression of ferritin in the caudoputamen and corpus callosum. Surprisingly, in animals injected with AAV-pDCX-eGFP we similarly observed two zones of hypointensity on T2* images. Cellular studies also showed the presence of mature (81.5%) and young neurons (6.1%) near the lateral ventricle in both postischemic and sham-operated animals, while macrophages in ischemic lesions were ferritin-positive (98.2%). (5) Conclusion: Ferritin overexpression induced by injection of AAV-pDCX-FerrH was detected by MRI using T2*-weighted images, which was confirmed by immunochemistry showing ferritin in young and mature neurons. Expression of eGFP also caused a comparable reduced MR signal intensity in T2*-weighted images. Additional studies are needed to investigate the potential and tissue-specific features of the use of eGFP and ferritin expression in MRI studies.

## 1. Introduction

Magnetic resonance imaging (MRI) is a highly-informative technique, which allows non-invasive visualization of anatomical structures and physiological processes, including neurogenesis, in the body [1,2,3,4,5,6,7,8,9,10,11,12,13,14,15,16,17,18,19]. Neurogenesis in pathological conditions is a complex process that evolves over time and includes migration of young neurons from neurogenic niches to the lesion site as well as formation of new neurogenic niches. Significant increases in neurogenesis were found in the animal models of brain ischemia and trauma [1,2,20,21,22]. This opens up new possibilities for therapy. Neurobiologists make assumptions about mechanisms of neural tissue plasticity based on ex vivo evaluations of the brain preparations obtained from animals at different stages of brain damage and restoration. Study of neurogenesis would greatly benefit from development of tissue-specific visualization probes.

To distinguish specific cell populations in brain tissue, these cells must be labeled with MRI-detectable probes. A variety of superparamagnetic iron oxide particles (SPIOs) are used as MRI-contrasting agents for the injection into the lateral ventricles, or into the brain parenchyma near to the neurogenic subventricular zone (SVZ) [3,4,5,6,12,13,15,16,17,18,19]. The major drawback of this technique is that such agents create MRI-contrast regardless of whether they are absorbed by the cells or remain as free particles resided in the tissue [1,13,19]. Moreover, SPIOs are absorbed not only by neuronal precursors (NPCs) but also by the other cells in the brain, i.e., by astrocytes, microglia, oligodendrocytes, and mature neurons [7,8]. Therefore, labeling specificity for particle-based imaging probes is very low. This can be resolved by the use of SPIOs, conjugated with antibodies, specific to the NSCs/neuroblasts surface antigens [9,10,16]. However, since a substantial proportion of neuroblasts die and are then consumed by microglia, the SPIOs released from the dead cells can be accumulated in microglia and microphages [1], misleading cell tracking with MRI.

Another way to create MRI contrast is to use MRI-reporters [14]. Different reporter proteins, including the iron storage protein, ferritin, in combination with different viral vectors, were proposed as genetically-based reporters, [23]. In some studies, lentiviral and adenoviral vectors, carrying the ferritin-complementary deoxyribonucleic acid(cDNA), were injected into the SVZ, leading to the ferritin synthesis in the migrating neuroblasts [11,14]. However, this approach still leads to the labeling of the variety of cell types besides NPCs [11].

This labeling specificity problem can be resolved by employing tissue-specific promoters that drive the gene reporter expression. Doublecortin (DCX) is the protein that is expressed within the brain, preferentially in neuroblasts and young neurons, and therefore is commonly considered as a marker of neurogenesis [21]. Several studies have used the doublecortin promoter (pDCX) for neuroblasts-specific transgene expression [24,25,26,27,28,29]. However, this approach was not applied to the MRI visualization of neurogenesis. The aim of this work was to explore if overexpression of ferritin, a nontoxic iron-binding protein, under the doublecortin promoter can be used for non-invasive visualization of neurogenesis in the rat brain using MRI.

## 2. Results

### 2.1. Neurological Deficit and Time Course of Ischemic Lesions

The neurological scores (NS) of animals after middle-cerebral-artery occlusion (MCAO) are presented in Table 1. After MCAO, animals of all groups with had severe neurological deficit on the first day after surgery, which slightly decreased by the end point of the observations. The groups showed similar NS at all time points and did not differ significantly. Sham-operated animals show no dysfunctions of the central nervous system at all time points.

**Figure 1:**
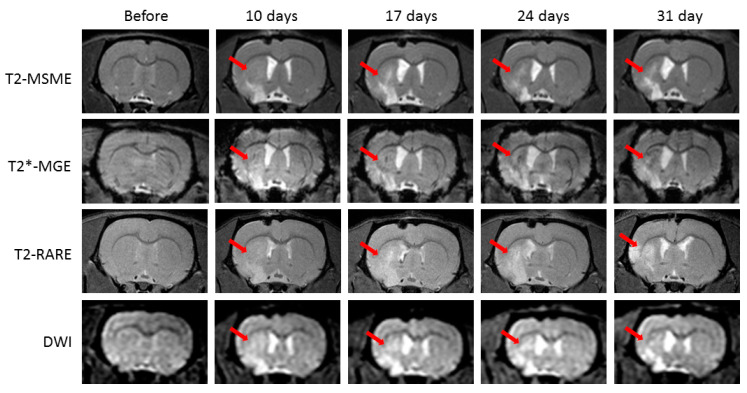
Example of the ischemic lesion evolution detected in the rat brain In vivo before middle-cerebral-artery occlusion (MCAO) and on days 10, 17, 24, and 31 after surgery and days 7, 14, 21, and 28 after phosphate-buffered saline (PBS) injection on T2-weighted multislice multiecho (T2-MSME), T2*-weighted multiple gradient echo (T2*-MGE), T2-weighted turbo rapid acquisition with relaxation enhancement (T2 TURBO RARE), and diffusion-weighted images (DWI). Red arrows point to the ischemic lesion area.

All animals subjected to MCAO surgery develop extensive brain lesions in the areas fed by the middle cerebral artery. Figure 1 shows evolution of the ischemic lesion in the rat brain after MCAO detected in MRI In vivo.

Ischemic lesions located in the caudoputamen and neocortex were clearly identified as bright areas (signal hyperintensity) on T2-, T2*-, and diffusion-weighted images (DWI). Ischemic lesion boundaries became clearer visible in the ipsilesioned cortex in two weeks following MCAO surgery. Signal intensity in the caudoputamen area did not increase overtime, even slightly decreased by the day 31 in the T2 and DWI. Changes in signal hyperintensity in the cerebral cortex on the T2*-weighted images showed similar dynamics. The caudoputamen area is bright in the ipsilateral hemisphere on T2- and T2*-weighted images due to transient ischemia, but it also includes a stable zone of signal hypointensity that appears on the day 10 and intensifies by the day 31.

### 2.2. Injections of Both AAV-pDCX-FerrH and AAV-pDCX-eGFP Cause Signal Hypointensity on T2*-MGE Images in the Sham-Operated and Post-Ischemic Animals

**Figure 2:**
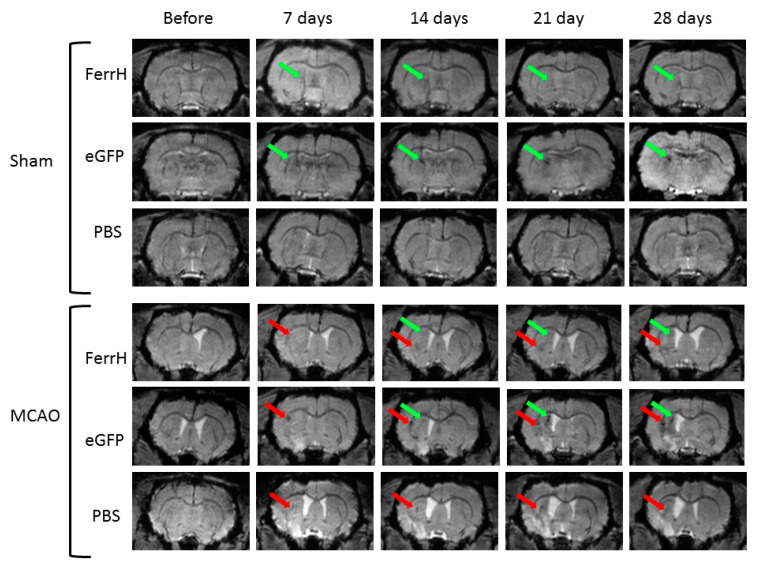
Evolution of the signal hypointensity zones detected in the rat brain In vivo before MCAO and on days 7, 14, 21, and 28 after injection of AAV-pDCX-FerrH, AAV-pDCX-eGFP or PBS using T2*-weighted MGE pulse sequence. Red arrows point to the signal hypointensity in the ischemic lesion area. Green arrows point to the signal hypointensity in the rat brain area close to the SVZ. Abbreviations: MCAO, middle-cerebral-artery occlusion; PBS, phosphate-buffered saline; AAV, adeno-associated viral backbone; pDCX, doublecortin promoter; FerrH, ferritin heavy chain; eGFP, enhanced green fluorescent protein.

The longitudinal changes of MRI signal intensity of the rat brain on T2*-weighted images are shown on the Figure 2.

Zones of hypointensity can be clearly seen near the left lateral ventricle and SVZ starting on the 7th day after injection in both FerrH-Sham and eGFP-Sham animals. This hypointensity became more expressed on days 14, 21, and 28 after viral injection. The phosphate-buffered saline (PBS) injection did not cause any changes on the T2*-weighted images on sham-operated animals. In MCAO-operated animals two zones of hypointensity are seen on T2*-weighted images in the FerrH-MCAO and eGFP-MCAO groups—one zone of hypointensity is located near the left lateral ventricle and SVZ; another zone of hypointensity can be seen inside the ischemic lesion. Both zones became clearly visible on days 14, 21, and 28 after viral injections. Interestingly, defined zones of signal hypointensity on T2* images in the brain of animals in the PBS-MCAO group are visible in the ischemic lesion (Figure 2, bottom row). Since no viral vectors were injected to those animals, the signal hyperintensity might be related to hemorrhage and accumulation of macrophages in the lesion.

**Figure 3:**
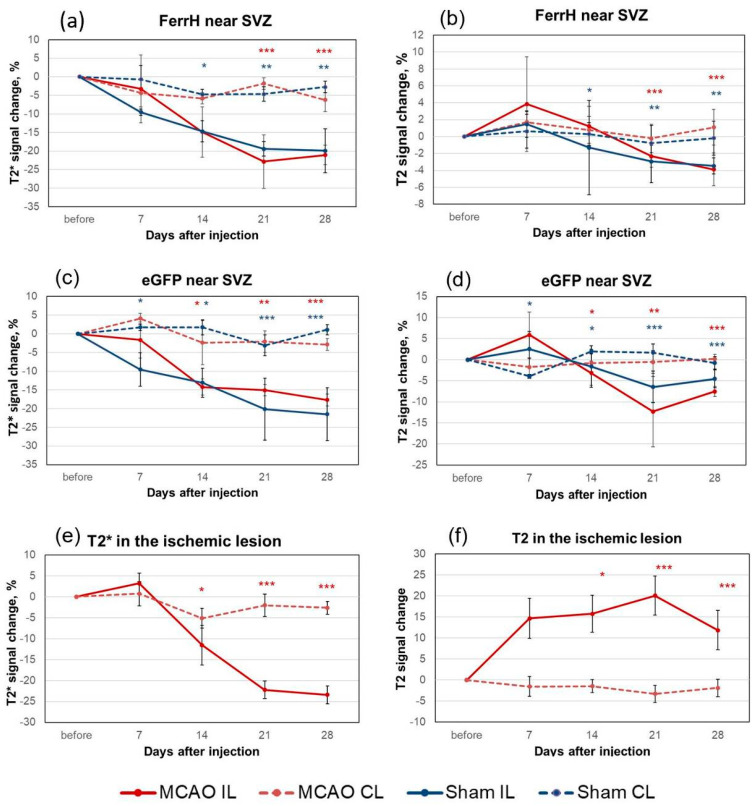
Quantification of the MRI signal in the zones of hypointensity on T2*- and T2-weighted images on different days after injection of AAV-pDCX-FerrH and AAV-pDCX-eGFP. (**a**,**b**) Percentage change of signal hypointensity near the SVZ on T2*- (**a**) and T2-weighted (**b**) images before and after AAV-pDCX-FerrH injection. Numbers are shown relative to the baseline (before MCAO or sham operation). (**c**,**d**) Percentage change of signal hypointensity near the SVZ on T2*- (**c**) and T2-weighted (**d**) images before and after AAV-pDCX-eGFP injection. (**e**,**f**) Percentage change of signal hypointensity areas in the ischemic lesion (FerrH and eGFP groups combined). Statistically significant differences in MRI signal change between ipsilateral and contralateral hemispheres were marked with stars (*), according to ANOVA after LSD’s correction for multiple comparisons: * *p* < 0.05; ** *p* < 0.01; *** *p* < 0.001. Abbreviations: MCAO, middle-cerebral-artery-occlusion; AAV, adeno-associated viral backbone; pDCX, doublecortin promoter; FerrH, ferritin heavy chain; eGFP, enhanced green fluorescent protein; SVZ, subventricular zone; IL, ipsilateral; CL, contralateral.

Quantification of the MRI signal in the zones of hypointensity near the SVZ showed differences in the signal dynamics between ischemic (ipsilateral) and non-ischemic (contralateral) hemispheres on T2- and T2*-weighted images (Figure 3).

Hypointensity changes near the SVZ in the FerrH-MCAO and eGFP-MCAO groups showed similar dynamics (solid lines in the Figure 3a–d). The T2 values in the ipsilesioned hemisphere in these groups showed a tendency to decrease but did not change significantly except for the 21-day point for the eGFP-MCAO group. In contrast, T2*-weighted images revealed significant hypointensity starting from the day 7 after the AAV-pDCX-eGFP injection and from the day 14 after the AAV-pDCX-FerrH injection (Figure 3a,c). These hypointensities in the zone near the lateral ventricle decreased by 21.06 ± 2.65% (FerrH-MCAO group) and 17.70 ± 1.64% (eGFP-MCAO group) on day 28 after viral injections in post-ischemic conditions, and 19.93 ± 5.94% (FerrH-sham group) and 21.53 ± 7.07% (eGFP-sham group) in sham-operated groups (Figure 3a–d). Signal intensity in contralateral hemispheres did not change (dotted lines in the Figure 3).

Signal intensity changes were also measured in the ischemic lesion on T2- and T2*-weighted images (Figure 2, red arrows). Resultant percentage changes in the AAV-pDCX-eGFP and AAV-pDCX-FerrH groups were almost identical, therefore were combined in the Figure 3e,f. T2 signal intensity in the lesion area increased after MCAO up to 20% on day 21, *p* < 0.001 (Figure 3f). There was no change in the T2 signal intensity in the contralateral hemisphere of the same animals. Signal intensity in the lesion area on T2*-weighted images had decreased significantly in the ipsilateral hemisphere after MCAO reaching 23% decrease by day 28 (Figure 3e), while no signal changes were observed in the contralateral hemisphere (*p* < 0.001).

### 2.3. T2* Signal Hypointensity Correlates with FerrH and eGFP Accumulation

**Figure 4:**
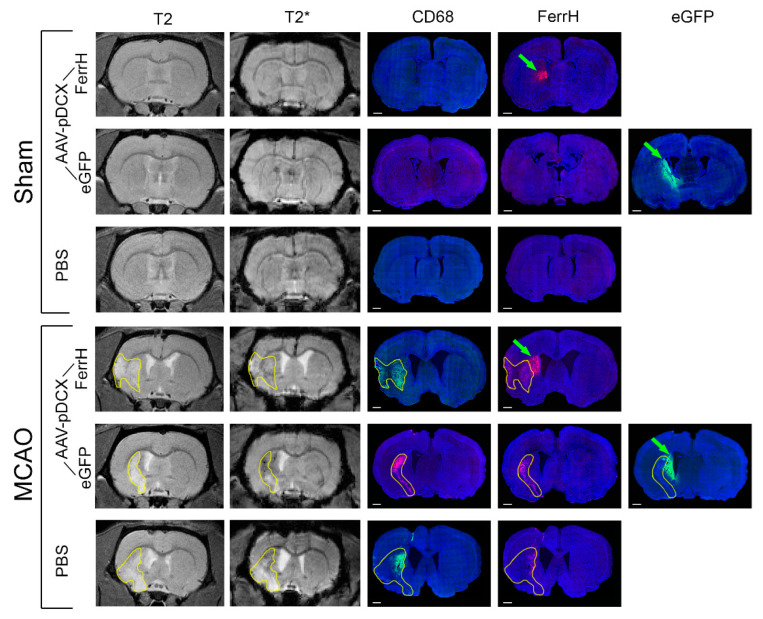
Representative T2- and T2*-weighted images in sham (top three rows) and MCAO (bottom three rows) animals are shown for day 28 after injection of viral vectors. Immunofluorescent staining were done for ferritin (FerrH) and activated microglia/macrophages (CD68). eGFP signal was read at the fluorescent microscope with a wavelength of 488 nm. Areas of ischemic lesion were delineated on T2-weighted and propagated to T2*-weighted and to the histological images (bottom three rows). Green arrows point to areas of the transgene expression near the SVZ outside of the ischemic lesion**.** Abbreviations: MCAO, middle-cerebral-artery occlusion; AAV, adeno-associated viral backbone; pDCX, doublecortin promoter; FerrH, ferritin heavy chain; eGFP, enhanced green fluorescent protein; PBS, phosphate-buffered saline; SVZ, subventricular zone.

To explore the origin of the signal hypointensity found in T2- and T2*-weighted MR images, a series of immunohistochemical staining was conducted to detect areas of ferritin, eGFP, and macrophage accumulation (Figure 4). Anatomically identical regions were identified in fluorescent-labeled micrographs of whole brain and slices in T2*-weighted images. In sham-operated animals (Figure 4, top three rows) the fluorescence of FerrH and eGFP showed a similar localization to the hypointensities on T2* images near the SVZ of the rat brain, while CD68 staining revealed no macrophages in the rat brain of the sham-operated animals, as expected.

After MCAO, zones of ischemic damage were clearly seen in the T2-weighted MRI as areas of signal hyperintensity (bright). The boundaries of ischemic lesion were delineated on T2-weighted and propagated to T2*-weighed and histological images (Figure 4, bottom three rows). Zones of signal hypointensities on T2* images were clearly visible inside of the bright ischemic lesion; those areas signal hypointensity and coincide with the areas of CD68/FerrH-positive cells in immunohistochemically stained fluorescent images. Unlike images from the PBS-MCAO group, both FerrH-MCAO and eGFP-MCAO groups show additional FerrH- and eGFP-positive areas near the lateral ventricle and SVZ. The locations of these areas corresponded to hypointensities on T2* images outside ischemic lesions.

**Figure 5:**
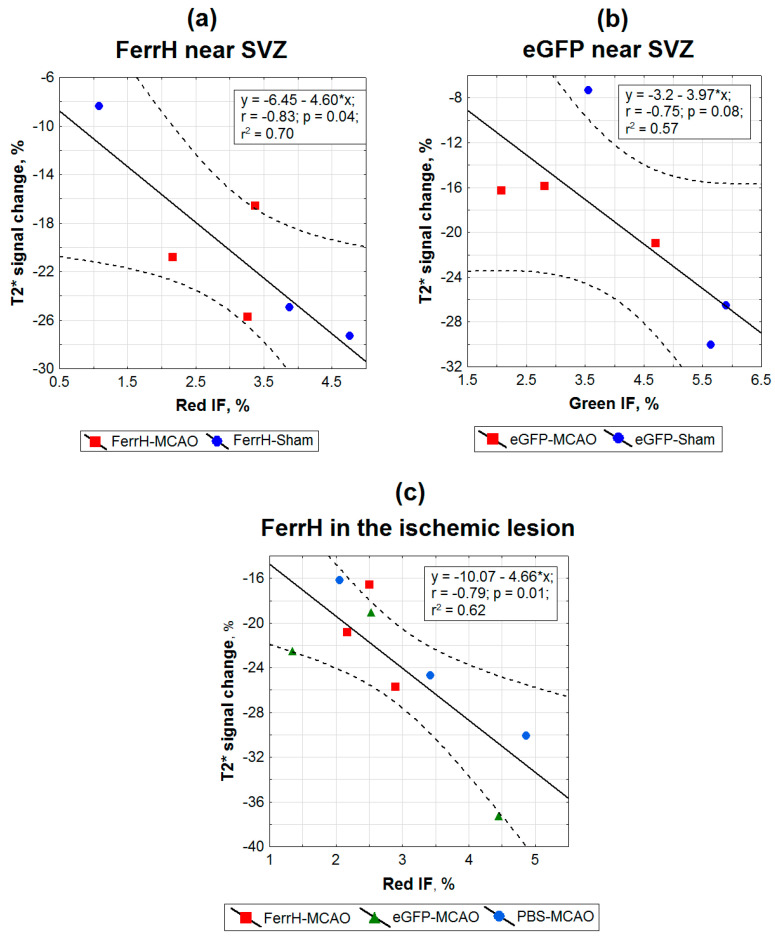
Histological validation of the MRI signal hypointensity areas at day 28 after AAV-pDCX-FerrH, AAV-pDCX-eGFP, or PBS with fluorescent images of eGFP signal, FerrH-, and CD68-stained brain sections. Panels (**a**–**c**) show linear regression plots of the percentage changes in T2* signal hypointensity relative to the baseline point as in the ischemic lesion relative to the symmetric contralateral anatomical region as a function of (**a**) FerrH IF in the zone near the SVZ, (**b**) eGFP IF in the zone near the SVZ, and (**c**) FerrH IF in the ischemic lesion. Points represent color-coded individual data for the animals from different groups. Abbreviations: MCAO, middle-cerebral-artery occlusion; AAV, adeno-associated viral backbone; pDCX, doublecortin promoter; FerrH, ferritin heavy chain; eGFP, enhanced green fluorescent protein; PBS, phosphate-buffered saline; SVZ, subventricular zone; red IF, intensity of red channel immunofluorescence in micrographs; green IF, intensity of green channel immunofluorescence in micrographs.

The relationships between percentage changes of T2* signal intensity and normalized fluorescent intensity (IF) of FerrH and eGFP in the same ROIs were assessed using linear regression analysis (Figure 5). The zones near the lateral ventricles and SVZ across animals from MCAO and sham groups were assessed separately for FerrH (Figure 5a) and eGFP IF (Figure 5b). For the zone located inside of the ischemic lesion, regression analysis was performed on the united data including animals of the FerrH-MCAO, eGFP-MCAO, and PBS-MCAO groups (Figure 5c). Statistically-significant correlation between T2* signal intensity and FerrH IF was found for the zones near the SVZ (r = 0.83, r^2^ = 0.70, *p* = 0.04) and inside of the ischemic lesion (r = 0.79, r^2^ = 0.62, *p* = 0.01). A correlation between percentage changes of T2* signal intensity and eGFP IF was close to significant (r = 0.75, r^2^ = 0.57, *p* = 0.08).

### 2.4. Iron Accumulation and Cellular Composition of FerrH- and eGFP-Positive Areas

**Figure 6:**
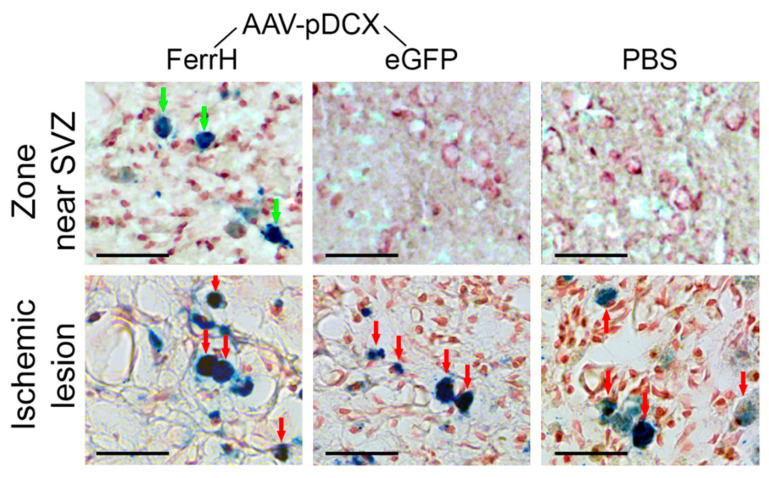
Perl’s Prussian blue histological staining for detection of iron accumulation in the zone near lateral ventricle and SVZ (top row) and in the ischemic lesion zone (bottom row). Green arrows point to the cells with iron accumulation in the zone near the SVZ; red arrows point to the cells with iron accumulation in the ischemic lesion. Scale bar corresponds to 50 µm. Abbreviations: AAV, adeno-associated viral backbone; pDCX, doublecortin promoter; FerrH, ferritin heavy chain; eGFP, enhanced green fluorescent protein; PBS, phosphate-buffered saline; SVZ, subventricular zone.

Iron accumulation in the animal brain tissue after MCAO or sham operation is shown in the Figure 6. Zones of iron accumulation were found in the ischemic lesion area in all groups independent of the injection type, FerrH, eGFP, or PBS (red arrows). Iron-accumulated cells in the lesion zone in all groups had a round shape. Outside the ischemic lesion area, iron accumulation was found near the lateral ventricle and SVZ but only for the animals injected with AAV-pDCX-FerrH (Figure 6, top row, green arrows). The iron-accumulated cells in this zone were identified as neurons with slightly elongated shape. Other studied groups did not show any iron accumulation outside ischemic lesion.

**Figure 7:**
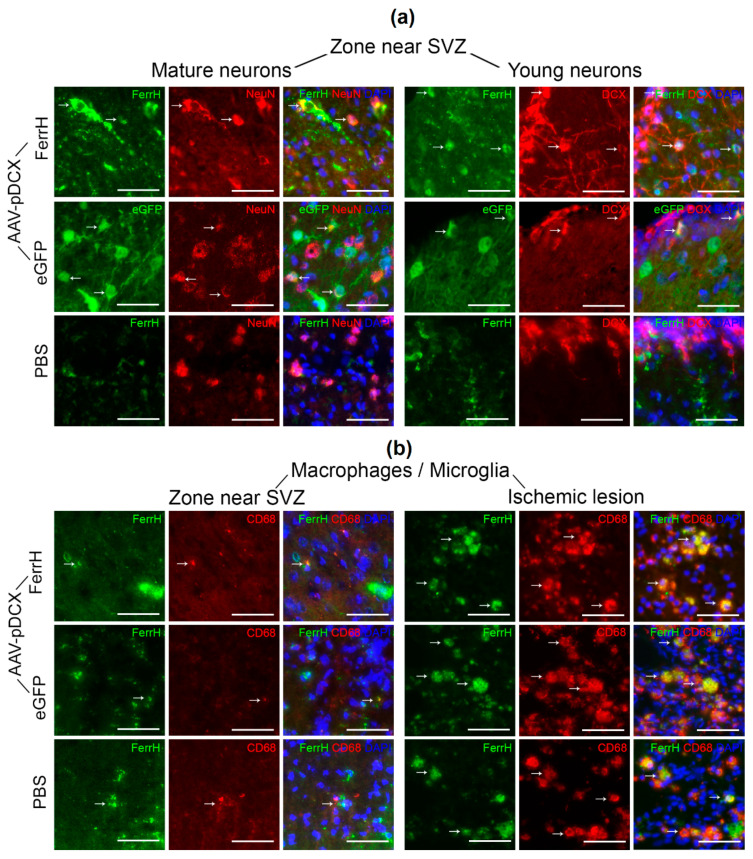
Examples of immunofluorescent staining for FerrH, NeuN (mature neurons), DCX (young neurons), and activated microglia\macrophages (CD68). (**a**) Double-immunofluorescence images stained for mature (FerrH\NeuN for the Ferr-MCAO and PBS-MCAO group, eGFP\NeuN for the eGFP-MCAO group), young (FerrH\DCX for the FerrH-MCAO and PBS-MCAO group, and eGFP\DCX for the eGFP-MCAO group) neurons in the zone near the lateral ventricle and SVZ. (**b**) Double-immunofluorescence images stained for microglia\macrophages (FerrH\CD68) in the near lateral ventricle and SVZ (left three rows) and inside the ischemic lesion (right three rows). For eGFP-MCAO group, FerrH is visualized by far-red fluorochrome and is shown as green pseudo color. Scale bars corresponds to 50 µm. Abbreviations: MCAO, middle-cerebral-artery occlusion; AAV, adeno-associated viral backbone; pDCX, doublecortin promoter; FerrH, ferritin heavy chain; eGFP, enhanced green fluorescent protein; PBS, phosphate-buffered saline; SVZ, subventricular zone.

Figure 7a illustrates typical colocalization of FerrH- and eGFP-positive cells with the markers of mature (NeuN) and young (DCX) neurons in animals after MCAO in the brain zone near the lateral ventricle and SVZ outside ischemic lesion. Similar types of colocalization was observed in sham-operated animals.

In animals in the FerrH-MCAO group, the bulk of the ferritin-expressing neurons were located near the lateral ventricle and SVZ, and only few neurons were found inside the ischemic lesion. Ferritin-expressing neurons have large euchromatin nuclei, which indicates the synthetic activity of DNA. Ferritin was localized in the cellular cytoplasm, in the axonal hillock, and in the axons, but is absent in the thinner neuronal branches. eGFP expression was found in the cytoplasm, axonal hillock, and in the axons including thinner neuronal branches. Ferritin clusters form conglomerates around the nucleus. The nuclei did not show the heterochromatization characteristic of karyopycnosis and the blabbs (vesicular protrusions and chromatin fragmentation) characteristic of karyorrhexis.

The density of mature ferritin-expressing neurons was lower in comparison with the density of mature neurons expressing eGFP. The eGFP-positive neurons formed large networks near the lateral ventricle and SVZ. In the eGFP-MCAO group, small number of mature neurons was observed inside the ischemic lesion. A large number of eGFP-positive mature neurons was found in the olfactory bulb both in animals after MCAO and in sham-operated animals. Small numbers of young neurons expressing ferritin or eGFP were located in the SVZ (Figure 7a, right panel), but the majority of ferritin- or eGFP-expressing cells near the SVZ were identified as mature neurons. Some ferritin- or eGFP-expressing cells were triple-positive (FerrH/DCX/NeuN or eGFP/DCX/NeuN).

**Figure 8:**
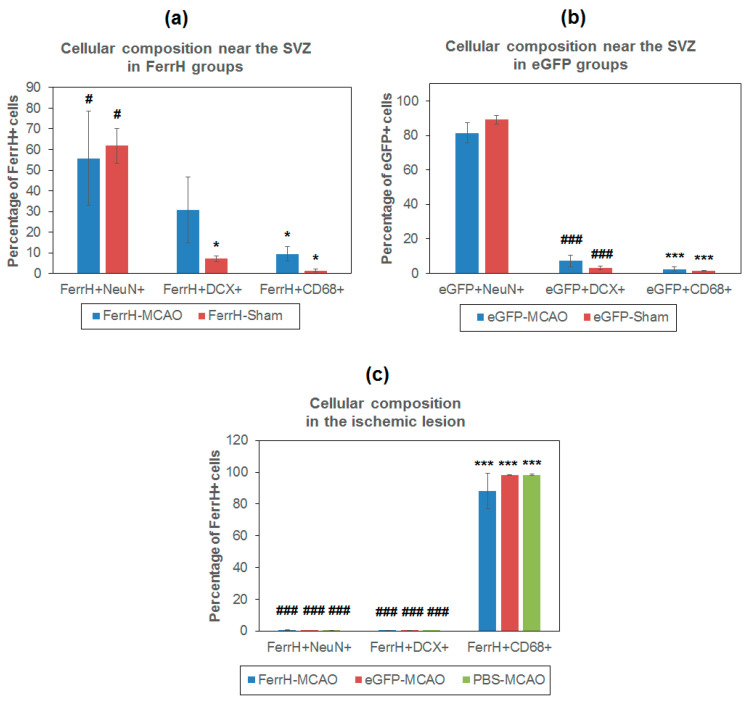
Cellular composition of two zones of MRI signal hypointensity in T2*-weighted images, near the SVZ and inside of the ischemic lesion area. (**a**) Percentage of mature (NeuN+) and immature (DCX+) neurons and activated microglia\macrophages (CD68+) in the zone near the lateral ventricle and SVZ in animals injected with AAV-pDCX-FerrH, (**b**) AAV-pDCX-eGFP, and (**c**) in the zone of ischemic lesion. Statistically-significant differences in percentage of cells according to ANOVA after LSD’s correction for multiple comparisons: in comparison with the percentage of NeuN+ cells,* *p* < 0.05 and *** *p* < 0.001; in comparison with the percentage of CD68+ cells, # *p* < 0.05 and ### *p* < 0.001. Abbreviations: MCAO, middle-cerebral-artery occlusion;, AV, adeno-associated viral backbone; pDCX, doublecortin promoter; FerrH, ferritin heavy chain; eGFP, enhanced green fluorescent protein; PBS, phosphate-buffered saline; SVZ, subventricular zone.

Quantification of FerrH/NeuN, FerrH/DCX, eGFP/NeuN, and eGFP\DCX double-positive cells is shown on the Figure 8. The number of ferritin-expressing mature neurons was higher than the number of young neurons in both the FerrH-MCAO and FerrH-Sham groups (statistically significant only for the FerrH-Sham group, *p* = 0.03). A similar tendency was observed in the groups with the AAV-pDCX-eGFP administration—the number of mature neurons was statistically-significantly higher compared to the number of young neurons in both the eGFP-MCAO and eGFP-In the sham groups, these differences were even more pronounced compared to animals injected with AAV-pDCX-FerrH.

Figure 7b and Figure 8a–c demonstrate substantial differences in the number of ferritin-accumulating macrophages/microglia between the zone near the SVZ and the zone of ischemic lesion. In the zone near the SVZ, a very small number of FerrH/CD68 double-positive cells was observed, while inside of the ischemic lesion, these cells occupy the large volume of the ischemic lesion. A similar cellular disposition was observed in all groups of animals that underwent MCAO, regardless of the type of vector or PBS administered. The cellular composition in the ischemic lesion differed significantly from the zone near the SVZ. In the zone near the SVZ, the number of ferritin/eGFP-accumulating mature neurons statistically significantly exceeded the number of macrophages/microglial cells, while in the zone of ischemic injury, on the contrary, the number of macrophages/microglial cells significantly exceeded the number of both mature and young ferritin/eGFP-accumulating neurons.

### 2.5. RT-PCR Results

**Figure 9:**
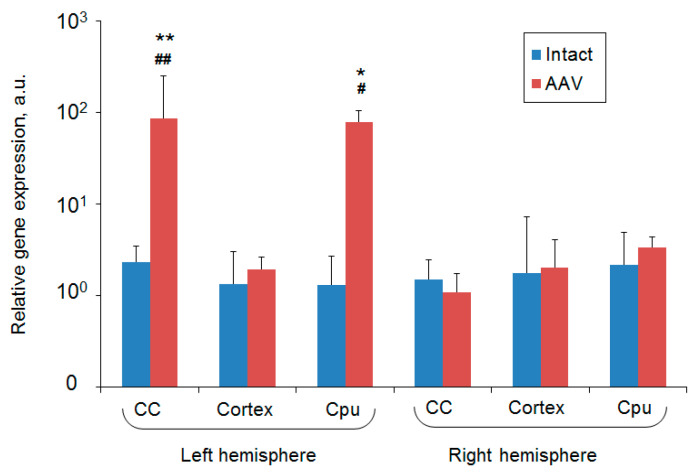
RT-PCR detection of ferritin mRNA expression in the different regions of the rat brain extracted from the intact (blue bars, n = 4) or AAV-pDCX-FerrH-injected animals (red bar, n = 4). Data are presented as mean ± SE. Statistically-significant differences in relative gene expression according to ANOVA after LSD’s correction for multiple comparisons: in comparison with probes of intact animals, * *p* < 0.05 and ** *p* < 0.01; in comparison with the right hemisphere, # *p* < 0.05 and ## *p* < 0.01. Abbreviations: CC, corpus callosum; CPu, caudoputamen; AAV, adeno-associated viral backbone.

RT-PCR data showed significant upregulated expression of the ferritin reporter gene in the corpus callosum and caudoputamen in the left hemisphere of the rat brain on day 7 after intracerebral injected of the adenoviral vector construct. No increase in ferritin mRNA expression was detected in the same brain structures extracted from the right hemisphere of the same animals or in the control (intact) group (Figure 9). These results are in correspondence with MRI findings—the areas of signal hypointensity of the rat brain injected with AAV-pDCX-FerrH were located in the caudoputamen and near the SVZ of the left hemisphere.

## 3. Discussion

The present work demonstrates the feasibility of longitudinal visualization of neurogenesis In vivo with MRI gene reporters expressed under the doublecortin promoter. We have shown the possibility of temporal analysis of neurogenesis in the same experimental animals at different time points after MCAO or sham surgery. The adult brain is now known to have dividing precursor cells that can make both glia and neurons. This the vast majority of published studies of the neurogenesis kinetics are still based on mitotic markers, such as bromodeoxyuridine (BrdU) [32,33]. Young neurons can be detected with the imaging probes expressed under the doublecortin promoter [24,27]. Some recent publications made attempts to follow neurogenesis In vivo using fluorescent, optical, or MRI-detectable probes [25,34]. The unique feature of the current study is a combination of MRI and fluorescent imaging probes expressed under the doublecortin promoter, specific to young neurons. The choice of the AAV–viral backbone is the not random; it is based on a previous publication by Vande Velde et al. [35] that showed reduced inflammatory response and as well as significantly-enhanced contrast to background on T2*-weighted MRI in the rodent brain if the imaging probes were delivered with AAV in comparison with lentiviral vectors. The major findings of the current study are the following.

1. Results of our study showed that rat brain could be successfully infected with AAV-pDCX-FerrH and AAV-pDCX-eGFP viral vectors for expression of either ferritin or eGFP. Both vectors caused at about 20% decrease in signal hypointensity in the areas near the SVZ on T2*-weighted MRI at one month after intracranial injection of the viral constructs.
2. The location of the signal hypointensity areas coincides with zones of ferritin and eGFP accumulation in immunohistochemical slides and zones of iron accumulation in Prussian blue staining a month after viral injection. RT-PCR data confirmed upregulated expression of the ferritin in the corpus callosum and caudoputamen in the left hemisphere of the rat brain on day 7 after intracerebral injected of the adenoviral vector construct.
3. The main source of the signal hypointensity near SVZs in the AAV-pDCX-FerrH and AAV-pDCX-eGFP groups are mature neurons with a small percentage of young neurons.
4. The main source of the signal hypointensity in the ischemic lesion area in AAV-pDCX-FerrH, AAV-pDCX-eGFP, and PBS-injected groups is macrophages.

Some questions remain to be answered. The first question that needs attention is MRI-signal specificity to young neurons. Despite the original intention being to label young neurons using a DCX promoter in the AAV vectors, the percentage of young neurons labeled with FerrH or eGFP detected near the lateral ventricle (SVZ of the rat brain) was small relative to the much larger number of probe-positive cells identified as mature neurons; specifically, 30.6% DCX-positive cells vs. 55.7% NeuN-positive cells in sham-operated animals and 7.6% DCX-positive cells vs. 61.8% NeuN-positive cells in the animals after MCAO. The higher percentage of cells expressing doublecortin in animals after MCAO is consistent with the results of many studies showing an increase in the production of new neurons after ischemia [21,36]. Based on the literature data, DCX expression occurs very early during neuronal differentiation, young neurons express doublecortin during first 2 weeks post-mitotic stage after maturation starts [37,38]. In our study, the origin of the ferritin-expressed cells was identified at a much later time point (28 days after viral injection) when the brains were fixed for histology. Therefore, it might be necessary to conduct the follow-up study with imaging and histology at early time points after viral infection. Another explanation might be neuronal plasticity; there is a possibility that young neurons that were originally infected with the ferritin or eGFP transgenes were continuously expressing those proteins even after maturation. There might also be a possibility of secondary transfection of mature cells; there was a report of transient doublecortin expression in mature neurons [39].

Secondly, zones of hypointensity were found not only near the SVZ in the rat brain but also inside of the ischemic lesion area. These zones of signal hypointensity became clearly visible starting on day 14 after viral injections using T2- and T2*-weighed MRI pulse sequences. Interestingly, defined zones of signal hypointensity in the ischemic lesion were visible in the brain of animals in the PBS-MCAO group where no viral vectors were injected. Precise histological examination revealed macrophages accumulation of in the lesion area. Prussian blue staining showed iron accumulation in macrophages, which is not surprising taking in consideration the fact that the key metabolic tasks of macrophages are cell recycling and free heme detoxification [40]. This brings additional questions regarding the specificity of the MRI signal hypointensity. Several other publications showed MRI signals from the macrophages accumulating iron [41,42]. The task of distinguishing the signal from the live cells expressing gene reporters and from macrophages metabolizing the iron-rich cells might be challenging. Based on our data, this might be partially accomplished with T2*-weighted images, since T2*- but not T2-weighted images show macrophage accumulation in the ischemic lesion. In the clinic, this might also be used as an additional marker for detection of inflammation after stroke.

Currently, neuroinflammation can be assessed with MRI using injected ultrasmall superparamagnetic particles of iron oxide (SPIO) that are absorbed by macrophages [43,44]. It is known that inflammation-activated microglia and macrophages accumulate ferritin [40], which, in turn, can be visualized using T2* MRI [4,5]. Although the T2* technique is successfully used in the clinic for detecting iron accumulation in Parkinson’s disease [45], Alzheimer’s disease [45], multiple sclerosis [45], and hemorrhagic stroke [46], there are no data on application of T2* for imaging neuroinflammation in ischemic stroke in human patients. In our study, as well as in the work of Carles et al. [47], iron accumulation by activated microglia/macrophages in ischemic lesion in rats can be revealed by signal hyperintensity on T2-weighted images in combination with signal hypointensity on T2*. In the clinic, this approach might be promising as a non-invasive biomarker of neuroinflammation after stroke without administration of exogenous SPIOs.

Thirdly, another surprising finding was that eGFP-transduced cells also caused MRI signal hypointensity on T2- and T2*-weighted images and the magnitude of the signal change was comparable with ferritin-expressed cells. eGFP is a widely used fluorescent marker that is easy to identify in ex vivo tissue slides or in live cells [48]. However, some of the published studies have similar to ours. In the publication of Vande Velde et al. [35,49] dark zones in the rodent brain were detected in T2*-weighted In vivo MRI at day 1, 1–2 weeks, 1 month, and 3 months after AAV- and LV-eGFP vector injections to the mouse brain [35]. The MRI hypointensity was related to the presence of infiltrated microglia/macrophages. In other reports, a dual reporter consisting of ferritin and eGFP produced image hypointensity in glioma cells [50] and for visualization of the neuronal network [49]. In the case of dual reporters, it might be hard to distinguish the impact of each gene reporter, ferritin or eGFP, on the resulted MRI signal. A study, published by the Pérez-Torres et al., described that the specific MRI pulse sequences based on a magnetization transfer (MT) effect in tissue are capable of detecting eGFP expression in vitro and In vivo in the mouse brain [51]. The above study reported that MT-MRI can be protein-specific and concentration-dependent. This opens up new opportunities in MRI sequence development/refinement for detection and quantification of gene-reporter expression. Based on our data, there was no iron accumulation in the eGFP-positive areas of the brain. Additional studies of the unexpected MRI sensitivity to eGFP expression are needed.

## 4. Materials and Methods

### 4.1. Vector Constructs

The human DCX promoter was selected from NCBI RefSeqGene NG_011750.1 (bases 3258-6766). Since the DCX promoter is quite large, first the Woodchuck hepatitis virus posttranscriptional regulatory element (WPRE) was removed from AAV-TF-CamKII0.4-eGFP, where CaMKIIα0.4 is a calmodulin-kinase-II promoter [52]. Next, to generate AAV-DCX-eGFP, the CamKII0.4-promoter was replaced by human DCX-promoter (pDCX) by inserting a two gBlocks (GenScript) into BspTI and Eco32I. Then, to create AAV-pDCX-FerrH, eGFP was replaced by a FerrH-gBlock using BamHI and SpeI. The sequence of this gBlock was based on the FerrH cDNA as described in Vande Velde et al. [35]. The resulting AAV-pDCX-eGFP and AAV-pDCX-FerrH transfer plasmids were used for the production of two recombinant adeno-associated viral vectors (rAAV) for expression of eGFP and FerrH, correspondingly.

The AAV vectors were produced by the Leuven viral vector core as describe previously [53]. DNase-resistant AAV particles were quality-controlled using QPCR to assess a physical titer for each production. AAV2/9_DCX-intron-eGFP had a titer of 3.02 × 10^12^ GC mL^−1^ and AAV2/9_DCX-intron-FerrH, a titer of 3.44 × 10^12^ GC mL^−1^. AAV vectors had a typical average yield of 5 × 10^11^ particles/mL.

### 4.2. Animals and Experimental Design

Adult Sprague–Dawley male rats (300–350 g) were bred in the specific pathogen-free (SPF) vivarium of the Institute of Pharmacology of the Siberian Branch of the Russian Academy of Science. Animals were housed in individually ventilated cages (one animal per cage, 10/14 light/dark cycle, temperature of 22 to 24 °C, humidity of 40 to 50%, water and SPF granulated chow ad libitum). The experimental protocol was approved by the Bioethical Committee of the Institute of Cytology and Genetics of the Siberian Branch of the Russian Academy of Sciences and the Bioethical Committee of the Biological Institute at Tomsk State University.

In total 26 animals were used in this study—18 animals for In vivo MRI and immunohistochemical analysis, another eight animals for RT-PCR. Nine animals were subjected to transient brain ischemia using the middle-cerebral-artery occlusion (MCAO) model; other nine animals were sham-operated. On 3rd day after the surgery animals were intracranially injected with AAV-pDCX-FerrH (n = 3), AAV-pDCX-eGFP viral vectors (n = 3), or PBS (n = 3). The same vectors or PBS were injected to the sham-operated group, three rats with each vector.

Animals were studied In vivo with MRI before MCAO or sham operation, and on days 7, 14, 21, and 28 after intracranial injection. Physiological variables including respiration rate, heart rate, and body temperature were monitored during the surgical procedure and MRI scanning. Neurological scores were assessed before each scanning. After the last MRI scan, animals were transcardially perfused with 4% paraformaldehyde (PFA) under ether anesthesia. Brains were removed and fixed overnight in PFA at 4 °C. The brains were then cryoprotected in a graded concentration of sucrose in phosphate buffer (24 h at 10% and 24 h at 20%) at 4 °C, frozen in liquid nitrogen, and stored at −80 °C prior to immunohistochemical staining.

### 4.3. MCAO Model

**Figure 10:**
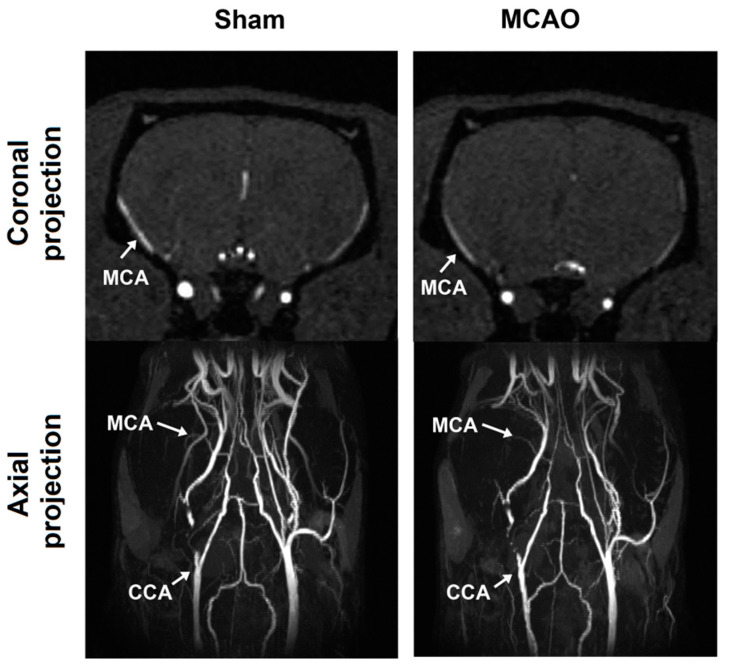
Representative images of magnetic resonance angiography in coronal (**top**) and axial (**bottom**) projections after sham and MCAO surgery. White arrows show the presence of blood flow in the common carotid artery (CCA) and middle cerebral artery (MCA).

Focal brain ischemia was achieved by the temporary occlusion of the middle cerebral artery with silicone-coated sutures (Doccol, Sharon, MA, USA), which is also known as the MCAO model [30]. The MCAO model was performed as described previously [54]. In brief, the filament was inserted into the circle of Willis through the external carotid artery to occlude the middle cerebral artery for 1 h. After that the filament was removed and blood flow was recovered. Recovery of cerebral blood flow immediately after surgery was monitored by magnetic resonance angiography (Figure 10) using a fast low-angle shot three-dimensional gradient echo (FLASH-3D GRE) pulse sequence with the following parameters: TE = 2.1 ms, TR = 15 ms, FA = 25°, FOV = 4 cm^3^, matrix size of 256 × 256 × 128, axial projection, scan time 6 min. The matrix was interpolated to a dimension of 256 × 256 × 256, 3D images were obtained using the maximum intensity projection (MIP) function in ParaVision 5.1 (Bruker Corporation, Billerica, MA, USA) and the intensity of the MR blood signal was compared to the contralateral side for the common carotid artery (CCA) and middle cerebral artery (MCA).

The animals from the control group underwent the same procedures except for filament insertion (sham operation). All surgeries were performed under isoflurane inhalation anesthesia (3% for induction and 1.5–2% for maintenance in air). Rectal temperature was monitored and maintained at the 37 ± 0.5 °C using a feedback-controlled heating blanket.

### 4.4. Viral Microinjections

For the intracerebral injections of the viral vectors or PBS, the animals were anesthetized in an induction chamber with inhalation of 4% isoflurane in air at a flow rate of 0.7 L/min; anesthesia was maintained during the surgery with 1–2% isoflurane in air using the laboratory vaporizer (Ugo Basile S.R.L., Gemonio, Italy). For the surgery and intracerebral injections, an animal was placed in a stereotactic frame Narishige SR-5R (Narishige Scientific Instrument Lab, Tokyo, Japan) that was custom-modified to enable fine movement of the injection syringe in the frontal and sagittal planes and to perform injections under certain angles. Eye gel “artificial tears” were used to keep rodent’s eyes moisturized during the surgery.

A midline incision of the skin was made to unbar the skull, a small hole was drilled in the skull using the bregma as a reference (stereotaxic zero) of the following stereotactic coordinates: anteroposterior 0, lateral 1.5 mm, and dorsoventral 5 mm, which corresponded to the of neurogenic subventricular zone (SVZ), based on the anatomical atlas of the rat brain [55]. The syringe for microinjections had a Hamilton needle (catalogue number 65460-04) equipped with 1-mm-diameter glass capillaries Narishige G-1 (Narishige Scientific Instrument Lab, Tokyo, Japan), stretched out at the day of injection using Narishige PC-10, (Narishige Scientific Instrument Lab, Tokyo, Japan). The needle with the glass capillary was inserted to a 4.67-mm depth in the rat brain tissues with a 18.7° tilt of the capillary to the middle line. The 1.5–2 µL vector injection of PBS was done during 10 min that allows slow-rate penetration of the injection solution to the brain tissue. Then the capillary was slowly removed from the brain during 1 min. Injection of the studied viral vectors or PBS was done to the left hemisphere of the brain. The incision was aseptically closed and covered with topical 10% synthomycin liniment (Nizhpharm, Nizhny Novgorod, Russia). The animal recovered on the warm pad. After the surgery, the animals were kept in individually ventilated cages.

### 4.5. In vivo MRI Studies

MRI experiments were performed using a 11.7 T Biospec small-animal MRI scanner (Bruker Corporation, Billerica, MA, USA) equipped with a transmit–receive volume coil 50 mm diameter. During MRI scans, animals were lightly anesthetized with 1.5–2% isoflurane in oxygen (1 L/min). The imaging protocol included the following pulse sequences:

(1) T2-weighted multislice multiecho (T2-MSME): TR = 2700 ms, TE = 7.3ms, FOV = 36 × 36 mm, image resolution 0.2 × 0.2 mm^2^, slice thickness 1 mm, matrix 180 × 180, 1 signal average, scan time 6 min 24 s.
(2) T2-weighted turbo rapid acquisition with relaxation enhancement (T2 TURBO RARE): TR = 2 s, TE = 7.6 ms, FOV = 36 × 36 mm, image resolution 0.12 × 0.12 mm^2^, slice thickness 1 mm, matrix 300 × 300, 3 signal averages, turbo factor 4, scan time 7 min 30 s.
(3) T2*-weighted multiple gradient echo (T2*-MGE): number of echoes = 9, first echo TE = 2.718 ms, echo spacing 2.9 ms, TR = 950 ms, flip angle 20 degree, FOV = 36 × 36 mm, slice thickness 1 mm, image resolution 0.2 × 0.2 mm^2^, matrix 180 × 180, 4 signal averages, scan time 8 min.
(4) Diffusion-weighted imaging (DWI): TR/TE = 3200/19 ms, 120 × 120 matrix, FOV = 36 × 36 mm^2^, matrix size = 128 × 128, section thickness = 0.9 mm, number of diffusion gradient directions = 6, one signal average, scan time 1 min 4 s.

Combinations of the different MRI techniques adds diagnostic value to In vivo imaging of the brain. Specifically, T2-weighted sequences are used for imaging of tissue edema and inflammation. Long T2 relaxation times of water-bound protons are used to generate a water-specific contrast resulting in high signal intensity of tissue fluid, acute swelling of cells and interstitial fluid accumulation. In the current study, two different T2-weighted MRI sequences were used for better visualization of the brain lesion. In the T2-MSME, a single composite radiofrequency pulse is used to collect all echoes in a train with the same phase encoding in several slices [56], while the T2-RARE MRI technique changes the phase-encoding gradient for each of the acquired echoes [57]. Using of both methods allow more reliable visualization of the tissue inflammation.

T2*-weighted MRI sequences are less sensitive to edema, and are mostly used for detection of deoxygenated hemoglobin, hemosiderin in lesions and tissues, and intracranial hemorrhage [58]. Signal-void areas on T2*-weighted images are most commonly used for detection of iron accumulation in cells overexpressing ferritin [23].

Diffusion-weighted imaging (DWI) is a form of MR imaging based upon measuring the random Brownian motion of water molecules within a voxel of tissue. Diffusion is routinely used in the clinic in detection of cerebral ischemia [59]. The combination of different imaging techniques was beneficial in the current study for precise evaluation of the extent of the ischemic damage to the rat brain and detection of the areas of ferritin and eGFP expression.

### 4.6. RT-PCR

Four rats received an intracerebral injection of AAV-pDCX-FerrH. The second group of three intact rats served as a control. Injection of the AAV-vector was done to the left hemisphere of the rat brain. On day 7 after injection, animals were decapitated under ether anesthesia. The brain was frozen in liquid nitrogen and kept at −80 °C. At the day of the PCR experiment, the brains were stepwise thawed at −18 °C, then at −4 °C, in saline. Cortex, corpus callosum, and striatum were cut out from the right and left hemispheres. Total RNA was isolated from each brain structure in 1 mL of the TRIzol reagent (Invitrogen, Carlsbad, CA, USA) according to the manufacturer’s instructions. The concentration and purity of RNAs were determined using the NanoDrop ND-2000 spectrophotometer (OD260/280, ~2.0) (Thermo Scientific, Waltham, MA, USA). Reverse transcription of 1 μg of RNA was performed using MMLV RT kit (Evrogen, Moscow, Russia). The ferritin cDNA was amplified with the following primers, forward (5′-CCCATTTGTGTGACTTCATT-3′) and reverse (5′-AGATATTCCGCCAAGCCA-3′), using qPCRmix-HS SYBRR kit (Evrogen, Moscow, Russia) and run in triplicate using on the LightCycler^®^ 480 II Real-Time PCR System (Roche, Basel, Switzerland). The thermal cycling conditions were as follows: initial denaturation at 95 °C for 1 min, 40 amplification cycles each consists of denaturation at 95 °C for 15 s, annealing at 60 °C for 10 s, and elongation at 72 °C for 30 s. The delta−delta CT method with normalization to mouse beta-actin expression [60] was used to calculate the relative expression of the human ferritin gene.

### 4.7. Immunochemistry and Histology

Coronal brain sections of 10 μm thickness were prepared using an HM525 cryostat (Thermo Fisher Scientific, Walldorf, Germany) at two brain locations: −1.58 mm (olfactory bulb) and + 0.74 mm (site of injection and ischemic lesion) from bregma, according to the mouse brain atlas [55]. To detect iron accumulation Perl’s Prussian blue histological staining was performed. The immunocytochemistry staining was done using the following primary antibodies: goat anti-doublecortin (Santa Cruz Biotechnology, Santa Cruz, CA, USA sc-8066, 1:100) to visualize immature neurons, rabbit anti-ferritin (Abcam, Cambridge, MA, USA, ab75973, 1:100) to visualize ferritin accumulation, rabbit anti-NeuN (MilliporeSigma, Burlington, MA, USA, ABN78, 1:200) to visualize mature neurons, and rabbit anti-CD68 (Wako Pure Chemical Industries, Richmond, VA, USA, 019-19741, 1:500) to visualize activated microglia. The following secondary antibodies were used: donkey anti-goat AlexaFluor594 (Jackson Immunoresearch, West Grove, PA, USA, 705-585-147, 1:500), donkey anti-rabbit AlexaFluor488 (Jackson Immunoresearch, West Grove, PA, USA, 711-545-152, 1:500), donkey anti-rabbit AlexaFluor594 (Jackson Immunoresearch, West Grove, PA, USA, 711-585-152, 1:500), donkey anti-goat AlexaFluor647 (Jackson Immunoresearch, West Grove, PA, USA, 703-605-155, 1:500), and donkey anti-rabbit AlexaFluor647 (Jackson Immunoresearch, West Grove, PA, USA, 711-605-152, 1:500). Double immunolabeling was used for FerrH and DCX, FerrH and NeuN, FerrH and GFAP, and FerrH and CD68. For double immunolabeling of brain sections of animals injected by AAV-pDCX-eGFP, secondary antibodies with dyes emission in red (AlexaFluor594) and far red (AlexaFluor647) part of spectra were used.

Sections were rinsed in PBS and incubated at room temperature overnight in PBS with block buffer solution and primary antibodies. After a triple rinse in PBS, sections were incubated with fluorescent secondary antibodies for 3 h at room temperature. Following a triple wash in PBS, sections were embedded in VECTASHIELD mounting medium (Vector Laboratories, Burlingame, CA, USA) with DAPI (40,6-diamidino-2-phenylindole). Fluorescent microscopy was performed using an Axio Imager Z2 microscope (Carl Zeiss, Oberkochen, Germany) with 10× objective and AxioVision 4.8 (Carl Zeiss, Oberkochen, Germany) software with a MozaiX module, which enables the creation of whole brain images by means of stitching together smaller images. Identical imaging parameters were set for all photographed sections.

### 4.8. Image Processing

**Figure 11:**
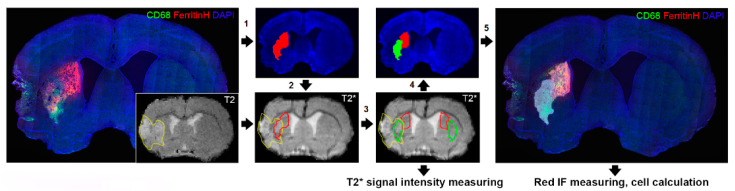
The processing steps in analysis of MRI and fluorescent images: (**1**) creating a binary image based on a fluorescent micrograph, (**2**) reducing the dimension of a binary image to the dimension of T2* image, (**3**) separation of brain zones based on the signal hyperintensity on T2-weighted images following by T2* signal intensity measurement, (**4**) scale-up regions of interest (ROIs) to measure IF and calculate labeled cells in micrographs (**5**).

All image analyses were performed using freely available ImageJ software (National Institutes of Health, Bethesda, MD, USA). T2*-weighted images were normalized by dividing each voxel value by the mean voxel value and co-registered to the endpoint (day 28). Figure 11 illustrates the steps for processing MRI and fluorescence images. Anatomically identical regions were identified in fluorescent-labeled micrographs of whole brain and slices in T2*-weighted images. Identical regions of interest (ROIs) for cell calculation in micrographs and signal measuring on T2*-weighted images were determined as follows: Initially, the resolution of the micrograph was reduced to the image resolution, combining with T2*-weighted image by transformation, and the Otsu thresholding method was applied [61]. An ROI was created from this binary image to measure signal intensity at all time points. A mirror ROI was used to measure signal intensity in the contralateral hemisphere. An average value of the signal intensity measured inside of the ROI on the T2*-weighted images at all time points in the ipsilateral and contralateral hemispheres, and the percentage changes in signal relative to the time point before surgery was calculated. Then, the ROI was scaled to the resolution of a micrograph and used for measuring of fluorescence intensity (IF) and cell calculation. Average values of green (eGFP) or red (FerrH) fluorescent signals were measured inside ROIs separately for the zones inside and outside of ischemic lesion within caudoputamen. Fluorescent signals were normalized using the highest value in all images [49]. The number and density of cells positive for ferritin, eGFP and double-positive for Ferr/NeuN, eGFP/NeuN, Ferr/DCX, eGFP/DCX, Ferr/CD68, and eGFP/CD68 was calculated on the microphotographic fluorescent images as well as the percentage of cells with co-expression of ferritin or eGFP with NeuN, DCX, and CD68.

### 4.9. Statistics

All statistical analyses were carried out in Statistica 10.0 for Windows (StatSoft Inc., Tulsa, OK, USA). A linear regression analysis was performed to find relations between the density of ferritin-expressing cells (by histology) and the percentage of MRI signal change in the same area. Differences were analyzed using two-way analysis of variance with repeated measurements (repeated measures ANOVA), taking into account the correction for the multiplicity of measurements, followed by post hoc least significant difference (LSD) tests. Linear regression analysis was conducted using the density of ferritin-positive cells as independent variable and MRI signal intensity in the corresponding brain zones as dependent variable. The statistical significance for all the analyses was less than 0.05.

## 5. Conclusions

Overexpression of the MRI gene reporter ferritin as well as fluorescent reporter eGFP under the doublecortin promoter can be used for non-invasive visualization of neurogenesis in the normal and ischemic rat brain using T2*-weighted MRI. Most of these cells are mature neurons. There is a possibility that young neurons that were originally infected with the viral vector for ferritin or eGFP production were continuously expressing those proteins even after maturation. In ischemic lesions, signal hypointensity in T2*-weighted images is caused by ferritin-expressing mature neurons as well as by macrophages accumulating ferritin. Follow-up studies at earlier time points after vector injection may address specificity problems of the detected signal from young neurons. The development of advanced imaging techniques can be useful for improving image contrast and separating the transgene signal from the inflammation-caused signal.

## Figures and Tables

**Table 1:** Animal neurological scores after surgery ^1^.

Surgery	Group	Total Number	Neurological Scores		
			1 Day	10 Days	31 Day
Sham-operated	AAV-pDCX-FerrH	3	0	0	0
Sham-operated	AAV-pDCX-eGFP	3	0	0	0
Sham-operated	PBS	3	0	0	0
MCAO	AAV-pDCX-FerrH	3	3 (1–4)	2 (0–3)	2 (0–3)
MCAO	AAV-pDCX-eGFP	3	3 (2–4)	2 (1–3)	2 (2–3)
MCAO	PBS	3	3 (3–4)	3 (2–4)	2 (2–3)

^1^ Neurological scores are presented as median (range). A score of 0 represents no dysfunction of the central nervous system; a score of 1 represents mild neurological deficit; a score of 2, moderate deficit; and a score of 3, severe deficit [30,31]. Abbreviations: MCAO, middle-cerebral-artery occlusion; PBS, phosphate-buffered saline; AAV, adeno-associated viral backbone; pDCX, doublecortin promoter; FerrH, ferritin heavy chain; eGFP, enhanced green fluorescent protein.

## Abbreviations

AAV	Adeno-associated viral backbone
ANOVA	Analysis of variance
BrdU	Bromodeoxyuridine
CC	Corpus callosum
CCA	Common carotid artery
cDNA	Complementary deoxyribonucleic acid
CD68	Cluster of differentiation 68
CPu	Caudoputamen
CT	Cycle threshold
DAPI	4′,6-diamidino-2-phenylindole
DCX	Doublecortin
DWI	Diffusion-weighted imaging
eGFP	Enhanced green fluorescent protein
FA	Flip angle
FerrH	Ferritin heavy chain
FLASH-3D	Fast low-angle shot three-dimensional
FOV	Field of view
GRE	Gradient echo
IF	Fluorescent intensity
LSD	Least significant difference
LV	Lentivirus
MCA	Middle cerebral artery
MCAO	Middle-cerebral-artery occlusion
MIP	Maximum intensity projection
MRI	Magnetic resonance imaging
MT	Magnetization transfer
MTC	Magnetization transfer contrast
NCBI	U.S. National Library of Medicine
NeuN	Neuronal nuclear antigen
NG2	Neural/glial antigen 2
NPCs	Neuronal precursors
NS	Neurological scores
OD	Optical density
PBS	Phosphate-buffered saline
pDCX	Doublecortin promoter
QPCR	Quantitative polymerase chain reaction
rAAV	Recombinant adeno-associated viral vectors
ROI	Region of interest
RT-PCR	Reverse-transcription polymerase chain reaction
SPF	Specific pathogen-free
SPIOs	Superparamagnetic iron oxide particles
SVZ	Subventricular zone
T2 TURBO RARE	T2-weighted turbo rapid acquisition with relaxation enhancement
T2*-MGE	T2*-weighted multiple gradient echo
T2-MSME	T2-weighted multislice multiecho
TE	Echo time
TR	Repetition time
WPRE	Woodchuck hepatitis virus posttranscriptional regulatory element

## References

[1] Mallett C.L., Shuboni-Mulligan D.D., Shapiro E.M. (2019). Tracking neural progenitor cell migration in the rodent brain using magnetic resonance imaging. Front. Neurosci..

[2] Manganas L.N., Zhang X., Li Y., Li Y., Hazel R.D., Smith D., Wagshul M.E., Henn F., Benveniste H., Djurić P.M. (2007). Magnetic Resonance Spectroscopy Identifies Neural Progenitor Cells in the Live Human Brain. Science.

[3] Vreys R., Velde G.V., Krylychkina O., Vellema M., Verhoye M., Timmermans J.P., Baekelandt V., Van der Linden A. (2010). MRI visualization of endogenous neural progenitor cell migration along the RMS in the adult mouse brain: Validation of various MPIO labeling strategies. NeuroImage.

[4] Granot D., Nkansah M.K., Bennewitz M.F., Tang K.S., Markakis E.A., Shapiro E.M. (2014). Clinically viable magnetic poly(lactide-co-glycolide) particles for MRI-based cell tracking. Magn. Reson. Med..

[5] Pothayee N., Cummings D.M., Schoenfeld T.J., Schoenfeld T.J., Dodd S., Cameron H.A., Belluscio L., Koretsky A.P. (2017). Magnetic resonance imaging of odorant activity-dependent migration of neural precursor cells and olfactory bulb growth. NeuroImage.

[6] Shuboni-Mulligan D.D., Chakravarty S., Mallett C.L., Wolf A.M., Forton S., Shapiro E.M. (2018). Age-dependent visualization of neural progenitor cells within the rostral migratory stream via MRI and endogenously labeled micron-sized iron oxide particles. bioRxiv.

[7] Nieman B.J., Shyu J.Y., Rodriguez J.J., Garcia A.D., Joyner A.L., Turnbull D.H. (2010). In vivo MRI of neural cell migration dynamics in the mouse brain. Neuroimage.

[8] Sumner J.P., Shapiro E.M., Maric D., Conroy R., Koretsky A.P. (2009). In vivo Labeling of Adult Neural Progenitors for MRI with Micron Sized Particles of Iron Oxide: Quantitation of Labeled Cell Phenotype. NeuroImage.

[9] Zhang F., Duan X., Lu L., Lu L., Zhang X., Zhong X., Mao J., Chen M., Shen J. (2016). In vivo Targeted MR Imaging of Endogenous Neural Stem Cells in Ischemic Stroke. Molecules.

[10] Zhong X.-M.M., Zhang F., Yang M., Wen X.-H.H., Zhang X., Duan X.-H.H., Shen J. (2015). In vivo Targeted Magnetic Resonance Imaging of Endogenous Neural Stem Cells in the Adult Rodent Brain. BioMed Res. Int..

[11] Iordanova B., Ahrens E.T. (2012). In vivo magnetic resonance imaging of ferritin-based reporter visualizes native neuroblast migration. NeuroImage.

[12] Granot D., Scheinost D., Markakis E.A., Papademetris X., Shapiro E.M. (2011). Serial Monitoring of Endogenous Neuroblast Migration by Cellular MRI. NeuroImage.

[13] Granot D., Shapiro E.M. (2014). Accumulation of micron sized iron oxide particles in endothelin-1 induced focal cortical ischemia in rats is independent of cell migration. Magn. Reson. Med..

[14] Vande Velde G., Raman Rangarajan J., Vreys R., Guglielmetti C., Dresselaers T., Verhoye M., Van der Linden A., Debyser Z., Baekelandt V., Maes F. (2012). Quantitative evaluation of MRI-based tracking of ferritin-labeled endogenous neural stem cell progeny in rodent brain. NeuroImage.

[15] Guglielmetti C., Praet J., Rangarajan J.R., Vreys R., De Vocht N., Maes F., Verhoye M., Ponsaerts P., Van der Linden A. (2014). Multimodal imaging of subventricular zone neural stem/progenitor cells in the cuprizone mouse model reveals increased neurogenic potential for the olfactory bulb pathway, but no contribution to remyelination of the corpus callosum. NeuroImage.

[16] Elvira G., García I., Benito M., Gallo J., Desco M., Penadés S., Garcia-Sanz J.A., Silva A. (2012). Live Imaging of Mouse Endogenous Neural Progenitors Migrating in Response to an Induced Tumor. PLoS ONE.

[17] Shapiro E.M., Gonzalez-Perez O., Manuel García-Verdugo J., Alvarez-Buylla A., Koretsky A.P. (2006). Magnetic resonance imaging of the migration of neuronal precursors generated in the adult rodent brain. NeuroImage.

[18] Panizzo R.A., Kyrtatos P.G., Price A.N., Gadian D.G., Ferretti P., Lythgoe M.F. (2009). In vivo magnetic resonance imaging of endogenous neuroblasts labelled with a ferumoxide-polycation complex. NeuroImage.

[19] Vreys R., Soenen S.J.H., De Cuyper M., Van Der Linden A. (2011). Background migration of USPIO/MLs is a major drawback for in situ labeling of endogenous neural progenitor cells. Contrast Media Mol. Imaging.

[20] Williamson M.R., Jones T.A., Drew M.R. (2019). Functions of subventricular zone neural precursor cells in stroke recovery. Behav. Brain Res..

[21] Nemirovich-Danchenko N.M., Khodanovich M.Y. (2019). New neurons in the post-ischemic and injured brain: Migrating or resident?. Front. Neurosci..

[22] Ramm P., Couillard-Despres S., Plötz S., Rivera F.J., Krampert M., Lehner B., Kremer W., Bogdahn U., Kalbitzer H.R., Aigner L. (2009). A Nuclear Magnetic Resonance Biomarker for Neural Progenitor Cells: Is It All Neurogenesis?. Stem Cells.

[23] Naumova A.V., Vande Velde G. (2018). Genetically encoded iron-associated proteins as MRI reporters for molecular and cellular imaging. Wiley Interdiscip. Rev. Nanomed. Nanobiotech..

[24] Karl C., Couillard-Despres S., Prang P., Munding M., Kilb W., Brigadski T., Plötz S., Mages W., Luhmann H., Winkler J. (2005). Neuronal precursor-specific activity of a human doublecortin regulatory sequence. J. Neurochem..

[25] Couillard-Despres S., Finkl R., Winner B., Ploetz S., Wiedermann D., Aigner R., Bogdahn U., Winkler J., Hoehn M., Aigner L. (2008). In vivo optical imaging of neurogenesis: Watching new neurons in the intact brain. Mol. Imaging.

[26] Marschallinger J., Schäffner I., Klein B., Gelfert R., Rivera F.J., Illes S., Grassner L., Janssen M., Rotheneichner P., Schmuckermair C. (2015). Structural and functional rejuvenation of the aged brain by an approved anti-asthmatic drug. Nat. Commun..

[27] Adamczak J., Aswendt M., Kreutzer C., Rotheneichner P., Riou A., Selt M., Beyrau A., Uhlenküken U., Diedenhofen M., Nelles M. (2017). Neurogenesis upregulation on the healthy hemisphere after stroke enhances compensation for age-dependent decrease of basal neurogenesis. Neurobiol. Disease.

[28] Wang X., Qiu R., Tsark W., Lu Q. (2007). Rapid promoter analysis in developing mouse brain and genetic labeling of young neurons by doublecortin-DsRed-express. J. Neurosci. Res..

[29] Walker T.L., Yasuda T., Adams D.J., Bartlett P.F. (2007). The doublecortin-expressing population in the developing and adult brain contains multipotential precursors in addition to neuronal-lineage cells. J. Neurosci..

[30] Longa E.Z., Weinstein P.R., Carlson S., Cummins R. (1989). Reversible middle cerebral artery occlusion without craniectomy in rats. Stroke.

[31] Bederson J.B., Pitts L.H., Tsuji M., Nishimura M.C., Davis R.L., Bartkowski H. (1986). Rat middle cerebral artery occlusion: Evaluation of the model and development of a neurologic examination. Stroke.

[32] Wojtowicz J.M., Kee N. (2006). BrdU assay for neurogenesis in rodents. Nat. Protoc..

[33] Bordiuk O.L., Smith K., Morin P.J., Semënov M.V. (2014). Cell proliferation and neurogenesis in adult mouse brain. PLoS ONE.

[34] Zhang F., Duan X., Lu L., Zhang X., Chen M., Mao J., Cao M., Shen J. (2017). In vivo Long-Term Tracking of Neural Stem Cells Transplanted into an Acute Ischemic Stroke model with Reporter Gene-Based Bimodal MR and Optical Imaging. Cell Transplant..

[35] Vande Velde G., Rangarajan J.R., Toelen J., Dresselaers T., Ibrahimi A., Krylychkina O., Vreys R., Van Der Linden A., Maes F., Debyser Z. (2011). Evaluation of the specificity and sensitivity of ferritin as an MRI reporter gene in the mouse brain using lentiviral and adeno-associated viral vectors. Gene Ther..

[36] Yamashita T., Ninomiya M., Hernandez Acosta P., Garcia-Verdugo J.M., Sunabori T., Sakaguchi M., Adachi K., Kojima T., Hirota Y., Kawase T. (2006). Subventricular Zone-Derived Neuroblasts Migrate and Differentiate into Mature Neurons in the Post-Stroke Adult Striatum. J. Neurosci..

[37] Ghosh H.S. (2019). Adult Neurogenesis and the Promise of Adult Neural Stem Cells. J. Exp. Neurosci..

[38] Ge W., He F., Kim K.J., Blanchi B., Coskun V., Nguyen L., Wu X., Zhao J., Heng J.I.T., Martinowich K. (2006). Coupling of cell migration with neurogenesis by proneural bHLH factors. Proc. Natl. Acad. Sci. USA.

[39] Hagihara H., Murano T., Ohira K., Miwa M., Nakamura K., Miyakawa T. (2019). Expression of progenitor cell/immature neuron markers does not present definitive evidence for adult neurogenesis. Mol. Brain.

[40] Nairz M., Theurl I., Swirski F.K., Weiss G. (2017). “Pumping iron”—How macrophages handle iron at the systemic, microenvironmental, and cellular levels. Pflugers Arch. Eur. J. Physiol..

[41] Amsalem Y., Mardor Y., Feinberg M.S., Landa N., Miller L., Daniels D., Ocherashvilli A., Holbova R., Yosef O., Barbash I.M. (2007). Iron-oxide labeling and outcome of transplanted mesenchymal stem cells in the infarcted myocardium. Circulation.

[42] Terrovitis J., Stuber M., Youssef A., Preece S., Leppo M., Kizana E., Schär M., Gerstenblith G., Weiss R.G., Marbán E. (2008). Magnetic resonance imaging overestimates ferumoxide-labeled stem cell survival after transplantation in the heart. Circulation.

[43] Albrecht D.S., Granziera C., Hooker J.M., Loggia M.L. (2016). In vivo Imaging of Human Neuroinflammation. ACS Chem. Neurosci..

[44] Saleh A., Schroeter M., Jonkmanns C., Hartung H.P., Mödder U., Jander S. (2004). In vivo MRI of brain inflammation in human ischaemic stroke. Brain.

[45] Stankiewicz J., Panter S.S., Neema M., Arora A., Batt C.E., Bakshi R. (2007). Iron in Chronic Brain Disorders: Imaging and Neurotherapeutic Implications. Neurotherapeutics.

[46] Wu G., Xi G., Hua Y., Sagher O. (2010). T2* Magnetic Resonance Imaging Sequences Reflect Brain Tissue Iron Deposition Following Intracerebral Hemorrhage. Transl. Stroke Res..

[47] Justicia C., Ramos-Cabrer P., Hoehn M. (2008). MRI detection of secondary damage after stroke: Chronic iron accumulation in the thalamus of the rat brain. Stroke.

[48] Li X., Wolf M.E. (2011). Visualization of virus-infected brain regions using a GFP-illuminating flashlight enables accurate and rapid dissection for biochemical analysis. J. Neurosci. Methods.

[49] Zheng N., Su P., Liu Y., Wang H., Nie B., Fang X., Xu Y., Lin K., Lv P., He X. (2019). Detection of neural connections with ex vivo MRI using a ferritin-encoding trans-synaptic virus. NeuroImage.

[50] Jiang C., Wu D., Haacke E.M. (2017). Ferritin-EGFP Chimera as an Endogenous Dual-Reporter for Both Fluorescence and Magnetic Resonance Imaging in Human Glioma U251 Cells. Tomography.

[51] Pérez-Torres C.J., Massaad C.A., Hilsenbeck S.G., Serrano F., Pautler R.G. (2010). In Vitro and In vivo Magnetic Resonance Imaging (MRI) Detection of GFP through Magnetization Transfer Contrast (MTC). NeuroImage.

[52] Gerits A., Vancraeyenest P., Vreysen S., Laramée M.-E., Michiels A., Gijsbers R., Van den Haute C., Moons L., Debyser Z., Baekelandt V. (2015). Serotype-dependent transduction efficiencies of recombinant adeno-associated viral vectors in monkey neocortex. Neurophotonics.

[53] Van Der Perren A., Toelen J., Carlon M., Van Den Haute C., Coun F., Heeman B., Reumers V., Vandenberghe L.H., Wilson J.M., Debyser Z. (2011). Efficient and stable transduction of dopaminergic neurons in rat substantia nigra by rAAV 2/1, 2/2, 2/5, 2/6.2, 2/7, 2/8 and 2/9. Gene Ther..

[54] Khodanovich M.Y., Kisel A.A., Akulov A.E., Atochin D.N., Kudabaeva M.S., Glazacheva V.Y., Svetlik M.V., Medvednikova Y.A., Mustafina L.R., Yarnykh V.L. (2018). Quantitative assessment of demyelination in ischemic stroke in vivo using macromolecular proton fraction mapping. J. Cereb. Blood Flow Metab..

[55] Paxinos G., Watson C. (2007). The Rat Brain in Stereotaxic Coordinates.

[56] Weaver J.B. (1988). Simultaneous multislice acquisition of MR images. Magn. Reson. Med..

[57] Hennig J., Nauerth A., Friedburg H. (1986). RARE imaging: A fast imaging method for clinical MR. Magn. Reson. Med..

[58] Chavhan G., Babyn P., Thomas B., Shroff M., Haacke M. (2009). Principles, Techniques, and Applications of T2*-based MR Imaging and Its Special Applications. Radiographics.

[59] Schaefer P.W., Grant E., Gonzalez G. (2000). Diffusion-weighted MR imaging of the brain. Radiology.

[60] Livak K.J., Schmittgen T.D. (2001). Analysis of relative gene expression data using real-time quantitative PCR and the 2^−ΔΔCT^ method. Methods.

[61] Otsu N. (1979). A threshold selection method from gray-level histograms. IEEE Trans. Syst. Man Cybern..

